# *Scutellaria baicalensis* in the Treatment of Hepatocellular Carcinoma: Network Pharmacology Analysis and Experimental Validation

**DOI:** 10.1155/2023/4572660

**Published:** 2023-02-23

**Authors:** Xin Cai, Shi Peng, Li Wang, Dongling Tang, Pingan Zhang

**Affiliations:** Department of Clinical Laboratory, Renmin Hospital of Wuhan University, 238 Jiefang Road, Wuhan 430060, China

## Abstract

**Objective:**

The aim of the study was to use a network pharmacological method and experimental validation to examine the mechanism of *Scutellaria baicalensis* (SB) against hepatocellular carcinoma (HCC).

**Methods:**

The traditional Chinese medicine systems pharmacology database and analysis platform (TCMSP) and GeneCards were used for screening of targets of SB for the treatment of HCC. Cytoscape (3.7.2) software was used to construct the “drug-compound-intersection target interaction” interaction network. The STING database was used to analyze the interactions of the previous intersecting targets. The results were visualized and processed by performing GO (Gene Ontology) enrichment analysis and KEGG (Kyoto Encyclopedia of Genes and Genomes) signaling pathway enrichment analysis at the target sites. The core targets were docked with the active components by AutoDockTools-1.5.6 software. We used cellular experiments to validate the bioinformatics predictions.

**Results:**

A total of 92 chemical components and 3258 disease targets including 53 intersecting targets were discovered. The results showed that wogonin and baicalein, the main chemical components of SB, could inhibit the viability and proliferation of hepatocellular carcinoma cells, promote apoptosis through the mitochondrial apoptotic pathway, and effectively act on AKT1, RELA, and JUN targets.

**Conclusion:**

SB has multiple components and targets in the treatment of HCC, providing possible potential targets for the treatment of HCC and providing a basis for further research.

## 1. Introduction

With roughly 906,000 new cases and 830,000 deaths from primary liver cancer worldwide in 2020, it is the sixth most prevalent cancer and the third main cause of cancer death globally, with typically three times as many men as women affected [1]. The main risk factors for hepatocellular carcinoma (HCC) are chronic infection with hepatitis B virus (HBV) or hepatitis C virus (HCV), which account for 56% and 20% of liver cancer deaths worldwide, respectively. Additionally, liver cancer can occur as a result of smoking, drinking excessive amounts of alcohol, being overweight, having type 2 diabetes, and eating food contaminated with aflatoxin. Since HCC starts insidiously and less than 30% of patients are suitable for radical treatment when first diagnosed, drug therapy is very important in the treatment of HCC [2]. Currently, some patients with hepatic insufficiency are not tolerant to first-line therapeutic agents and the treatment methods are expensive, which causes physical and psychological stress to these patients. Therefore, finding new effective drugs is an urgent matter to be addressed at present.

Many Chinese traditional herbs can have specific efficacy in disease treatment, but the specific mechanism of action is not clear. Network pharmacology has enabled some herbal components to be explored and studied, allowing more specific components to be discovered and studied, and the combination with molecular docking technology is beneficial to discover new drug targets and active drug components [3]. For example, Tu discovered artemisinin in *Artemisia annua* and thus effectively acted on malaria patients [4]. Previously, it was thought that “one drug for one target,” but modern research has demonstrated that the action of drugs in the body is far more complex than previously thought and that one drug may act on multiple targets and one target may be acted on by multiple drugs, and this idea of one drug for one target is no longer applicable to today's drug research [5]. Cyber pharmacology searches for key components of potential drug target combinations and facilitates drug research, especially anticancer drug research [6].

*Scutellaria baicalensis* (SB) has been used in China for thousands of years and was recorded in Shennong ben cao jing (The Classic of Herbal Medicine) as early as 200 AD, where it was used to treat liver and lung problems and wind-cold fever [7, 8]. Modern research has found that SB has antitumor effects on a variety of cancer cells, such as prostate cancer and head and neck tumors [9, 10]. The treatment of dividing cells with SB induces DNA damage and leads to cell death but does not cause cell mutation, which is a major side effect of conventional anticancer drugs [11]. This implies that SB has the potential to be a novel antitumor therapeutic agent.

**Figure 1:**
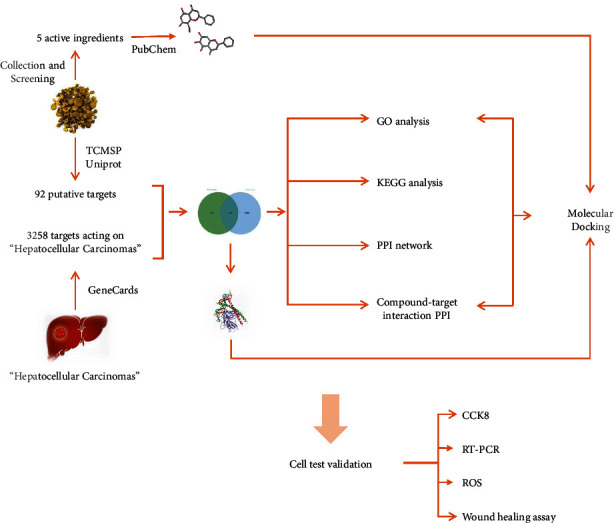
Conceptual framework of this study.

This study determined the primary active ingredients in SB for the treatment of HCC based on network pharmacology and obtained its potential targets. This research established the molecular mechanism of SB and supported it with cytological tests, giving SB a rationale for use in the treatment of HCC. The overall structure flowchart is shown in Figure 1.

## 2. Materials and Methods

### 2.1. Acquisition of Active Constituents and Targets of SB

The chemical components of SB were searched by the traditional Chinese medicine systems pharmacology database and analysis platform (TCMSP) (https://tcmspw.com/tcmsp.php). Oral bioavailability (OB) and drug likeness (DL) were used as the screening criteria, and chemical components with OB ≥ 30% or DL ≥ 0.18 were selected.

### 2.2. Screening of the Action Targets of SB

The corresponding targets of the obtained compounds were collected in the TCMSP database, and the obtained targets were subjected to gene name transformation and specification in the UniProt database (https//www.uniprot.org/).

### 2.3. Acquisition of Targets for Hepatocellular Carcinoma

We searched the GeneCards (https://www.genecards.org/) database for genes related to hepatocellular carcinoma using the keyword “Hepatocellular Carcinomas” and obtained the targets related to hepatocellular carcinoma after deduplication.

### 2.4. Screening of Targets of SB for the Treatment of Hepatocellular Carcinoma

Using Venny2.1 (https://www.liuxiaoyuyuan.cn/), the targets of SB component action and the targets related to hepatocellular carcinoma were intersected, and the overlapping targets were the targets related to the possible action of SB in hepatocellular carcinoma.

### 2.5. Constructing Drug-Compound-Intersection Target Interaction Networks

Cytoscape (3.7.2) software was used to construct the “drug-compound-intersection target interaction” interaction network. The nodes in the network diagram represent the disease, drug, active ingredients, and targets, respectively. The interactions between active ingredient and target, target and disease, or target and drug were represented by the connection of edges. A network analysis, a plug-in of Cytoscape, was used to analyze the topological properties of the network, and the network was arranged according to the degree value to obtain a network graph that can characterize the relationships between drugs, diseases, ingredients, and targets.

### 2.6. Protein-Protein Interaction (PPI) Network Construction

The STING database (https://string-db.org/) was used to analyze the interactions of the previous intersecting targets, import the target information into the database, select multipleproteins, set the species to *Homo sapiens*, set the confidence level to 0.9, hide the scattered nodes, and obtain the protein-protein interaction of the intersecting target genes. The protein-protein interaction (PPI) network of intersecting target genes was obtained. Cytoscape 3.7.0 software was applied to visualize the results, and topological analysis was performed with the aid of CytoNCA plug-in. The core targets were calculated and visualized with the median values of the parameters of mediator centrality, proximity centrality, degree centrality, eigenvector centrality, local average connectivity, and network centrality as the filtering conditions, respectively. These six parameters represent the topological importance of each individual node in the whole network, and the larger their quantified values, the more important the node is in that network.

### 2.7. GO Functional Annotation and KEGG Pathway Enrichment Analysis

The results were visualized and processed by performing GO (Gene Ontology) enrichment analysis and KEGG (Kyoto Encyclopedia of Genes and Genomes) signaling pathway enrichment analysis at the target sites, and the differences were considered statistically significant at *P* < 0.05. We use Sangerbox online processing tool (https://www.sangerbox.com/), a comprehensive and interactive friendly platform for clinical bioinformatics analysis. They used SpringCloud framework, java 11,10.2.30-MariaDB to build the website, website backend, and database. R 3.6.3, R 4.0.1, Perl, and JavaScript languages were applied to write and convert to arithmetic and analytical scripts.

### 2.8. Molecular Docking Study of Target Proteins and Active Components of SB

We download the mol2 format file of the 2D structure of the screened key compound from the PubChem database (https://pubchem.ncbi.nlm.nih.gov/), import it into AutoDockTools-1.5.6 software, check its spatial structure for errors, add the atomic charges, and assign the atomic types; all flexible keys are changed to default rotatable and saved in the PDBQT format as docking ligands. We select the core target of the previous screening, look for the 3D concept of the active ingredient in the UniProt database (https://www.uniprot.org/), enter the query PDB id into the PDB database (https://www.rcsb.org/), download the 3D structure of its corresponding protein, and save it in the PDB format. We import this 3D structure into AutoDockTools-1.5.6 software, perform water molecule removal, hydrogen addition, charge addition, and various optimizations, then set the grid box (bird box) as the default value and save it, and finally save the optimized protein in the PDBQT format as a pair of acceptors. According to the coordinates of the ligand in the target protein and the target protein activity pocket that had been set, molecular docking was performed using AutoDock Vina 1.1.2 to obtain the results, and the obtained results were imported into PyMOL software for visualization of the results.

### 2.9. Cell Culture and Drug Treatment

The HepG2 cell line was employed in cell experiments of this study. Cells were cultured in DMEM (Gibco, USA) containing 10% fetal bovine serum (Gibco, USA) at 37°C with 5% CO_2_ and 95% humidity. Wogonin and baicalein (WinHerb, CN) were dissolved in DMSO, and the drug solutions of 0.65 *μ*M, 1.25 *μ*M, 2.5 *μ*M, 5 *μ*M, and 10 *μ*M were configured.

### 2.10. CCK8 Assay

Cell viability was detected by employing the CCK8 assay (Meilunbio, CN). HCC cells in the logarithmic growth phase were seeded in 96-well plates, and 3 replicate wells were taken from each group. Cells were grown adherent and then replaced, and having taken 0.625∼10 *μ*M as a concentration gradient, the cells were treated with wogonin and baicalein. After 24 hours, 10 *μ*l CCK8 solution was added to each well. The absorbance at a wavelength of 450 nm was measured after 1 hour using a microplate reader.

### 2.11. RNA Extraction and qPCR

The mRNA level of key target molecules was detected by qPCR. The cells were seeded in six-well plates and pretreated with wogonin and baicalein for 24 h, respectively. Total RNA was extracted with Trizol (Vazyme, CN), and cDNA was synthesized by a reverse transcription reagent (Vazyme, CN). The internal reference group was GAPDH, and the results were expressed as 2^−ΔΔCt^. The previous experiments were repeated three times independently. The sequences of primers were as follows: Jun forward, 5′-ACTCGGACCTCCTCACCT-3′ and Jun reverse, 5′-CGTTGCTGGACTGGATTAT-3′; RELA forward, 5′-AGAGCAGCGTGGGGACTA-3′ and RELA reverse, 5′-ATGGGATGAGAAAGGACAGG-3′; AKT1 forward, 5′-CAAGGTGATCCTGGTGAA-3′ and AKT1 reverse, 5′-CGTGGGTCTGGAAAGAGT-3′.

### 2.12. ROS Assay

The intracellular ROS levels were measured using a reactive oxygen species assay kit (Beyotime Biotechnology, China). Briefly, the cells were seeded in 6-well plates and exposed to wogonin and baicalein for 24 h. Following the treatment, the cells were incubated with DCFH-DA for 20 min at 37°C and then observed using Olympus IX71 fluorescence microscopy (Japan).

### 2.13. Data Analysis

Statistical analysis was performed with GraphPad Prism 6.0. Image processing is conducted by image *J* and Sanger box 3.0. All data were presented as the mean ± SD. The one-way analysis of variance was applied for comparisons between the groups, and *P* < 0.05 indicated that the difference was statistically significant.

## 3. Results

### 3.1. Main Active Components and Their Targets of SB

A total of 105 compounds was founded in SB by TCMSP search, and 36 active ingredients were obtained after screening. The basic information about the active ingredients of SB is shown in Table 1. The gene names of the action targets were collected through the UniProt database, and the invalid and duplicate genes were deleted, and a total of 92 action targets of active ingredients of SB were obtained.

### 3.2. Target Screening of SB for the Treatment of HCC

**Figure 2:**
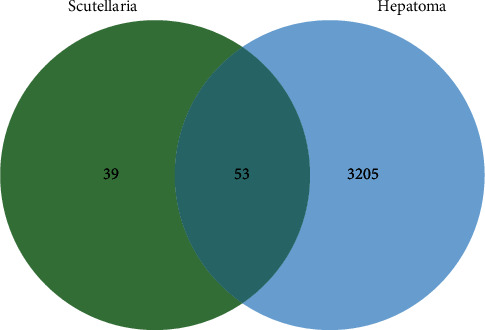
Venn diagram showing the numbers of the overlapping genes between SB and HCC.

The keyword “Hepatocellular Carcinomas” was used to collect 3,258 genes for related diseases in the GeneCards database. The screened 92 baicalin targets were put into the online website Venny2.1 to take the intersection with 3,258 primary liver cancer targets, and a total of 53 common targets were collected (Figure 2).

### 3.3. “Drug-Compound-Intersection Target” Analysis

**Figure 3:**
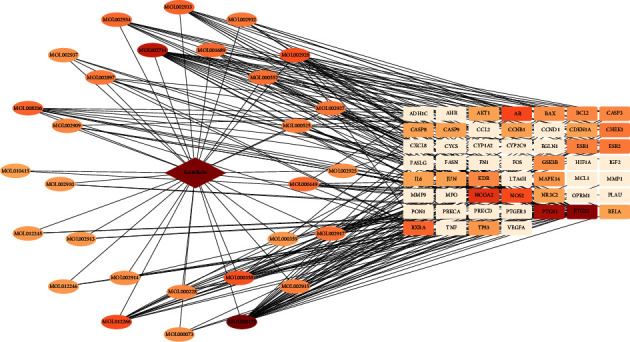
Interaction network shows the “drug-compound-intersection target.”

The “drug-compound-intersection target” interaction network is shown in Figure 3. In the figure, the central diamond node represents SB, the peripheral oval node represents the SB active ingredient, the number on the node is the corresponding TCMSP number of the compound, and the rectangle represents the “drug-disease” intersection target. “The color shades of the active ingredient nodes represent the number of targets connected to them. As seen in Figure 3, the network includes 83 nodes and 230 edges. Among the active ingredients of SB, the top five in the degree value are wogonin (30), baicalein (21), rivularin (14), beta-sitosterol (13), and oroxylin A (13), and the top five protein targets were PTGS2, (27) PTGS1, (23) NCOA2 (17), AR (15), and NOS2 (14). We subsequently selected wogonin and baicalein for the corresponding cytological experiments.

### 3.4. Construction and Analysis of Target PPI Network

**Figure 4:**
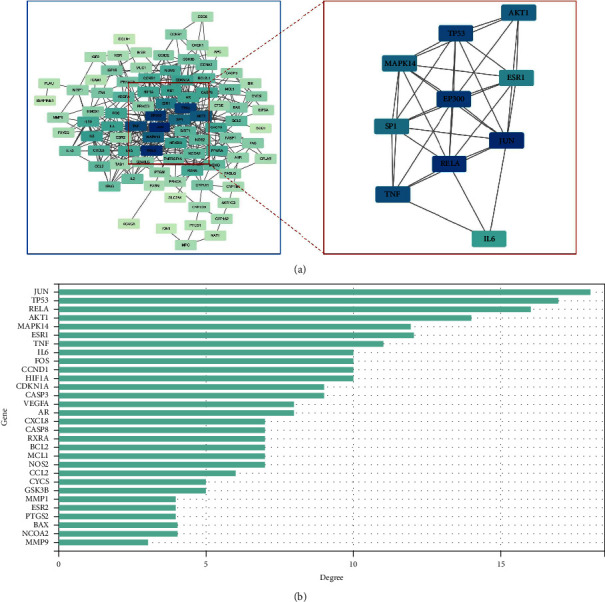
Interaction network of the overlapping targets. (a) The PPI network of the overlapping targets. (b) Bar plot of the number of hub gene links.

The 53 intersecting targets were imported into the STRING online data platform, the species genus was selected as *Homo sapiens,* the confidence level was set to 0.95, and the PPI graph was plotted (Figure 4(a)), where the nodes represent proteins and the edges represent protein-protein interactions. Bar graphs were made and analyzed to obtain the top 30 PPI core genes (Figure 4(b)) as follows: JUN, TP53, RELA, AKT1, MAPK14, ESR1, TNF, IL6, FOS, CCND1, HIF1A, CDKN1A, CASP3, VEGFA, AR, CXCL8, CASP8, RXRA, BCL2, MCL1, NOS2, CCL2, CYCS, GSK3B, MMP1, ESR2, PTGS2, BAX, NCOA2, and MMP9.

### 3.5. Key Signaling Pathways of SB in the Treatment of HCC

**Figure 5:**
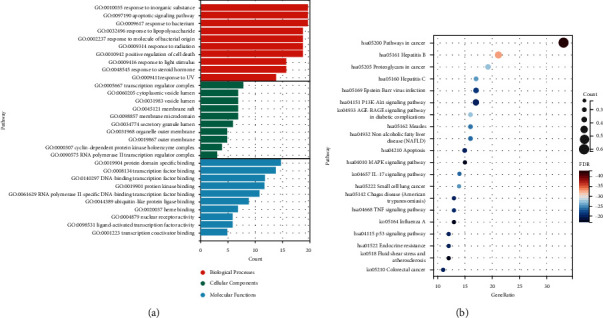
(a) GO enrichment analysis of the overlapping targets; (b) KEGG enrichment analysis of the overlapping targets.

To analyze the cellular components, molecular functions, and biological processes enriched in the 53 intersecting targets, we performed GO analysis on the intersecting targets, and the results showed that the key targets of SB for hepatocellular carcinoma were enriched in a total of 1,199 GO entries, including 1,087 bioinformatic processes (BP), 25 cellular components (CC), and 87 molecular functions (MF). The BP results suggest that it may be related to response to inorganic substance, apoptotic signaling pathway, and response to bacterium. The CC results suggest that it may be related to transcription regulator complex, cytoplasmic vesicle lumen, and vesicle lumen. The MF results suggest that it may be related to protein domain specific binding, transcription factor binding, and DNA-binding transcription factor binding.  Figure 5(a) shows the results of the top 10 GO analysis for BP, CC, and MF.

KEGG analysis of key targets of SB for hepatocellular carcinoma showed that the key targets were enriched in a total of 238 pathways, including those in various cancers, such as small cell lung cancer and colorectal cancer, involving hepatitis B, hepatitis C, Epstein–Barr virus, influenza A and PI3K-Akt signaling pathway, AGE-RAGE signaling pathway in diabetic complications, IL-17 signaling pathway, TNF signaling pathway, and p53 signaling pathway. The bubble plots are sorted by the gene ratio, the higher the number of genes in the top pathway, the larger the corresponding bubble in the plot, as shown in Figure B. JUN, RELA, and AKT1 were ranked in the top 5 of the core genes of the PPI network, which indicated that JUN, RELA, and AKT1 might be the key targets of SB for the treatment of hepatocellular carcinoma.

### 3.6. Molecular Docking

**Figure 6:**
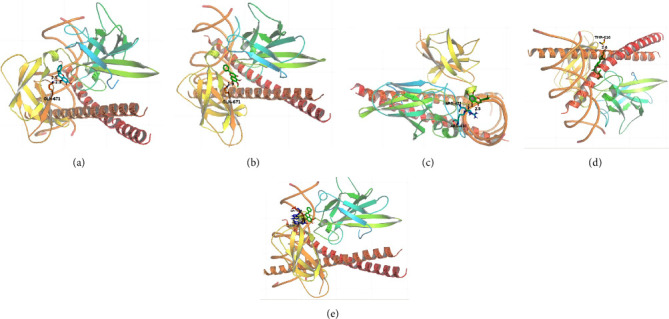
Results of molecular docking. (a) Action mode of baicalein with target JUN; (b) action mode of oroxylin A with target JUN; (c) action mode of rivularin with target JUN; (d) action mode of *β*-sitosterol with target JUN; (e) action mode of wogonin with target JUN.

We used the molecular docking technique to investigate the interaction between the main active components of SB and JUN, RELA, and AKT1 proteins. The stability of the binding between the receptor and the ligand depends on the binding energy. If the binding energy is less than −5 kJ/mol, it indicates that the target compound has some binding activity with the compound, and the lower the binding energy, the more stable the binding conformation of the receptor and the ligand. As shown in Table 2, baicalein, oroxylin A, rivularin, wogonin, and *β*-sitosterol and JUN, RELA, and AKT1 are used as examples to analyze their possible modes of action with receptors (Figure 6).

### 3.7. Wogonin and Baicalein Affects Cell Morphology and Proliferation

**Figure 7:**
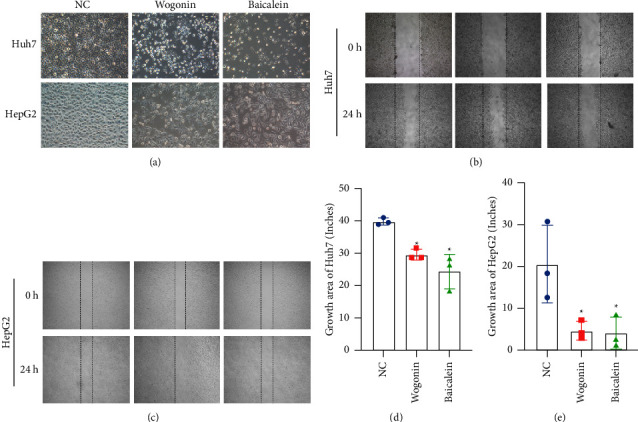
Cell morphology and cell scratches. (a) Cellular state changes in Huh7 cells and HepG2 cells after treatment with drugs; (b) Huh7 cell scratch results; (c) HepG2 cell scratch results; (d) growth area of Huh7 cells; (e) growth area of HepG2 cells. ^*∗*^*P* < 0.05, compared with the NC group.

To verify the effect of the active ingredient of SB on the growth of HepG2 cells and Huh7 cells, we compared the morphology of HepG2 cells and Huh7 cells in the blank group and the group treated with wogonin and baicalein. As shown in Figure 7(a), the cells in the blank group are in good growth condition, with the cells growing against the wall, clearly outlined, closely arranged, and proliferating at a faster rate. When treated with the drug for 24 h, a significant decrease in the number of cells was observed by microscopy, and the cells were crumpled and deformed and their proliferation was inhibited, as shown in Figure 7(a). The cell scratch width of the drug-treated group was significantly larger than that of the blank control group after 24 h. We assumed that the drug inhibits the proliferation of HepG2 cells and Huh7 cells, as shown in Figures 7(b)–7(e).

### 3.8. Wogonin and Baicalein Exerts an Inhibitory Role in the Viability of HCC Cells

**Figure 8:**
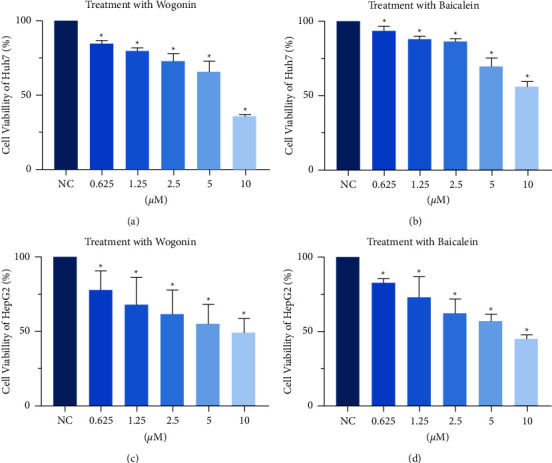
CCK8 assay analyzes the viability of HCC cells. (a) Cell viability of Huh7 cells after being treated with wogonin. (b) Cell viability of Huh7 cells after being treated with baicalein. (c) Cell viability of HepG2 cells after being treated with wogonin. (d) Cell viability of HepG2 cells after being treated with baicalein. ^*∗*^*P* < 0.05, compared with the NC group.

To further validate the effect of wogonin and baicalein on HCC cells, different concentrations (0∼10 *μ*M) of wogonin and baicalein were employed to act on HepG2 cells and Huh7 cells. After 24 hours, the effect of wogonin and baicalein on HepG2 cells and Huh7 cell viability were then observed. The CCK8 results demonstrated (Figure 8) that wogonin and baicalein at different concentrations had varying degrees of inhibitory effect, and with the increase of wogonin and baicalein concentration, the inhibitory effect on the cell viability of HCC cells was more significant.

### 3.9. Wogonin and Baicalein May Act on HCC Cells through Reactive Oxygen Species

**Figure 9:**
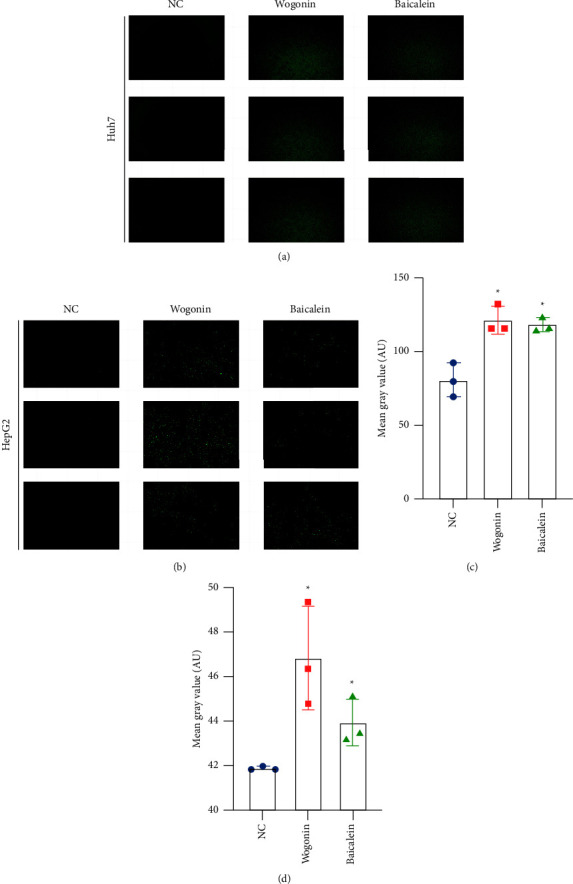
ROS levels of HCC cells. (a) ROS levels of Huh7 cells; (b) ROS levels of HepG2 cells; (c) statistical graph of ROS levels of Huh7 cells; (d) statistical graph of ROS levels of HepG2 cells. ^*∗*^*P* < 0.05, compared with the NC group.

To further investigate by what way wogonin and baicalein induce apoptosis, we used a reactive oxygen species assay kit to detect intracellular reactive oxygen species (ROS) levels, as shown in Figure 9, which were significantly higher than in the native control groups under the effect of the drug.

### 3.10. Wogonin and Baicalein May Act on JUN, RELA, and AKT1

**Figure 10:**
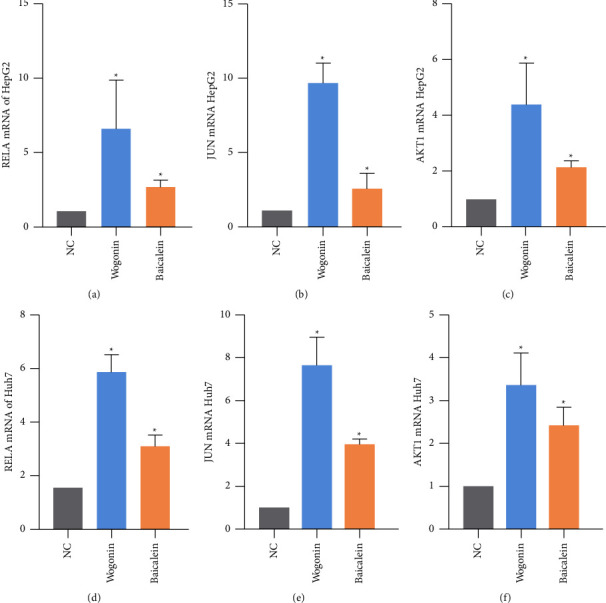
RELA, JUN, and AKT1 mRNA levels of HepG2 and Huh7 cells. (a) RELA mRNA levels of HepG2; (b) JUN mRNA levels of HepG2; (c) AKT1 mRNA levels of HepG2; (d) RELA mRNA levels of Huh7; (e) JUN mRNA levels of Huh7; (f) AKT1 mRNA levels of Huh7. ^*∗*^*P* < 0.05, compared with the NC group.

To verify the targets obtained from bioinformatic analysis, we used qPCR to detect the expression of JUN, RELA, and AKT1 mRNA after 24 h of drug treatment and found that JUN, RELA, and AKT1 mRNAs were significantly higher than those of the untreated group, as shown in Figure 10.

## 4. Discussion

HCC is a common cancer with extremely high incidence and mortality rates worldwide, and patients are often diagnosed at an advanced stage. Surgical treatment options are not applicable to these advanced HCC patients, making the development of new drugs critical for patients with advanced disease [12, 13]. Chinese herb plays an important role in the treatment of HCC, and it can improve the therapeutic effect together with chemotherapy drugs. Chinese herbs can successfully treat clinical symptoms and enhance patients' quality of life, according to clinical practice [14–16]. SB, a Chinese herb, is commonly used in the treatment of HCC. But further research is needed to understand how it treats HCC. In this study, the main components and targets of the action of SB in HCC were explored using network pharmacology and cytology experiments.

Network pharmacology is an emerging paradigm to systematically reveal the multicomponent and network regulation of prescriptions and has been widely applied to the screening of bioactive compounds and the study of efficacy mechanisms of various Chinese medicine prescriptions [17]. We determined that wogonin and baicalin are the primary active ingredients in SB in this investigation. We screened the SB potential therapeutic targets and liver cancer-related targets, and then we took the intersection to get 53 common targets that represent potential SB-HCC-therapy targets. We mapped PPI and examined key genes such as JUN, TP53, RELA, AKT1, MAPK14, ESR1, TNF, IL6, FOS, and CCND1 using STRING analysis of protein interactions. We enriched the common target genes in the GO pathway and KEGG pathway and found that AKT1, RELA, and JUN are likely to be potential therapeutic targets for hepatocellular carcinoma. AKT1 and AKT2 are widely distributed in the liver, and a recent study demonstrated that AKT1 and AKT2 mice are more susceptible to liver cancer [18]. Mice deficient in Akt1 and Akt2 exhibit impaired liver regeneration and increased mortality [19]. Xu et al. demonstrated that the mTORC2-Akt1 cascade pathway plays an important role in the development of HCC [20]. RELA, also known as p65, was shown by Murray to attenuate the proliferation of pancreatic ductal adenocarcinoma cells and prolong the survival time of mice [21]. JUN is the oncogenic transcription factor [22]. JNK/c-JUN is a recognized proapoptotic and proinflammatory pathway, and Lauricella et al. demonstrated that silencing of c-JUN reduced the susceptibility of HepG2 cells to apoptosis [23]. The results of the present experiment showed that AKT1 mRNA, RELA mRNA, and JUN mRNAs were significantly upregulated in HepG2 cells in the presence of two active components of SB, which is consistent with the previous findings. Our analysis of the GO pathway and KEGG pathway revealed that the main mechanisms of action of the active ingredients of SB were enriched in the PI3K-AKT signaling pathway, the MAPK signaling pathway, and the IL-17 signaling pathway. Several studies have demonstrated that the PI3K/AKT/mTOR pathway is a classical dysregulated pathway involved in the pathogenesis of HCC [24]. Moon and Ro demonstrated that the MAPK/ERK signaling pathway is often activated in HCC [25]. Ma et al. demonstrated that IL-17A is a tumor-promoting factor that regulates alcoholic fatty liver, liver fibrosis and HCC [26]. The conclusions from this study by bioinformatics were consistent with the previous results, and the analysis showed activation of the apoptotic pathway after drug treatment. The results of the cellular assay showed a decrease in cellular activity with increasing drug concentration. Further investigation of the cause of apoptosis revealed an increase in intracellular ROS levels after drug treatment, and it was hypothesized that the active ingredient of SB may cause apoptosis through the mitochondrial pathway. Previous studies have demonstrated that wogonin induces apoptosis in hepatocellular carcinoma cells through intracellular H_2_O_2_ production and Ca^2+^ release [27], Wang et al. demonstrated that baicalein induces apoptosis and autophagy in hepatocellular carcinoma cells through endoplasmic reticulum stress [28], and Huang et al. demonstrated that wogonin induces apoptosis by regulating PI3K-AKT signaling in breast cancer cells [29]. There is also a recent study demonstrating that wogonin inhibits the invasive effect of hepatocellular carcinoma cells through matrix metalloproteinase-9 [30]. This is consistent with the findings of the present study. Although some preliminary findings were obtained in the present study, further experimental validation is needed.

## 5. Conclusion

In this study, we investigated the drug-component-target-disease interactions of SB in the treatment of HCC using network pharmacology. SB may induce apoptosis in HCC cells by modulating the PI3K-AKT signaling pathway through the mitochondrial pathway. The findings of this study shed light on SB's anticancer properties and probable biological pathways in HCC and lay the groundwork for future research into SB or its active constituents as a viable adjunctive therapy to SB treatment.

## Figures and Tables

**Table 1:** The active ingredients of *Scutellaria baicalensis*.

Mol ID	Molecule names	OB (%)	DL
MOL001689	Acacetin	34.97	0.24
MOL000173	Wogonin	30.68	0.23
MOL000228	(2R)-7-hydroxy-5-methoxy-2-phenylchroman-4-one	55.23	0.2
MOL002714	Baicalein	33.52	0.21
MOL002908	5,8,2′-trihydroxy-7-methoxyflavone	37.01	0.27
MOL002909	5,7,2,5-tetrahydroxy-8,6-dimethoxyflavone	33.82	0.45
MOL002910	Carthamidin	41.15	0.24
MOL002911	2,6,2′,4′-tetrahydroxy-6′-methoxychaleone	69.04	0.22
MOL002913	Dihydrobaicalein	40.04	0.21
MOL002914	Eriodictyol (flavanone)	41.35	0.24
MOL002915	Salvigenin	49.07	0.33
MOL002917	5,2′,6′-trihydroxy-7,8-dimethoxyflavone	45.05	0.33
MOL002925	5,7,2′,6′-tetrahydroxyflavone	37.01	0.24
MOL002926	Dihydrooroxylin A	38.72	0.23
MOL002927	Skullcapflavone II	69.51	0.44
MOL002928	Oroxylin a	41.37	0.23
MOL002932	Panicolin	76.26	0.29
MOL002933	5,7,4′-trihydroxy-8-methoxyflavone	36.56	0.27
MOL002934	NEOBAICALEIN	104.34	0.44
MOL002937	DIHYDROOROXYLIN	66.06	0.23
MOL000358	Beta-sitosterol	36.91	0.75
MOL000359	Sitosterol	36.91	0.75
MOL000525	Norwogonin	39.4	0.21
MOL000552	5,2′-dihydroxy-6,7,8-trimethoxyflavone	31.71	0.35
MOL000073	Ent-epicatechin	48.96	0.24
MOL000449	Stigmasterol	43.83	0.76
MOL001458	Coptisine	30.67	0.86
MOL001490	bis[(2S)-2-ethylhexyl] benzene-1,2-dicarboxylate	43.59	0.35
MOL001506	Supraene	33.55	0.42
MOL002879	Diop	43.59	0.39
MOL002897	Epiberberine	43.09	0.78
MOL008206	Moslosooflavone	44.09	0.25
MOL010415	11,13-eicosadienoic acid, methyl ester	39.28	0.23
MOL012245	5,7,4′-trihydroxy-6-methoxyflavanone	36.63	0.27
MOL012246	5,7,4′-trihydroxy-8-methoxyflavanone	74.24	0.26
MOL012266	Rivularin	37.94	0.37

**Table 2:** Results for molecular docking.

Characteristics	PDB id	Active ingredients	Side length of the docking site	Docking coordinates	Combined energy (kcal/mol^−1^)
AKT1	2uzs	Baicalein	40	*x* = 20.035	−7.2
AKT1		Oroxylin A			−6.1
AKT1		Rivularin			−5.6
AKT1		Wogonin		*y* = 0.360	−6.7
AKT1		*β*-sitosterol		*z* = 8.631	−7.2
JUN	1s9k	Baicalein	40	*x* = 28.874	−7.8
JUN		Oroxylin A			−7.8
JUN		Rivularin		*y* = 28.619	−7.2
JUN		Wogonin		*z* = 60.875	−7.2
JUN		*β*-sitosterol			−7.5
RELA	1nfi	Baicalein	40	*x* = −5.372	−7.4
RELA		Oroxylin A			−7.1
RELA		Rivularin			−6.9
RELA		Wogonin		*y* = 65.520	−7.1
RELA		*β*-sitosterol		*z* = 45.295	−6.8

## Data Availability

The data used and analyzed during the current study are available from the corresponding author upon reasonable request.
